# Application of Plasma Levels of Olanzapine and *N*-Desmethyl-Olanzapine to Monitor Clinical Efficacy in Patients with Schizophrenia

**DOI:** 10.1371/journal.pone.0148539

**Published:** 2016-02-05

**Authors:** Mong-Liang Lu, Yi-Xiu Wu, Chun-Hsin Chen, Pei-Ting Kuo, Yi-Hua Chen, Chia-Hui Lin, Tzu-Hua Wu

**Affiliations:** 1 Department of Psychiatry, Taipei Medical University-Wan Fang Hospital, Taipei, Taiwan; 2 Department of Psychiatry, School of Medicine, College of Medicine, Taipei Medical University, Taipei, Taiwan; 3 Department of Clinical Pharmacy, School of Pharmacy, College of Pharmacy, Taipei Medical University, Taipei, Taiwan; 4 School of Public Health, College of Public Health and Nutrition, Taipei Medical University, Taipei, Taiwan; Benito Menni Complejo Asistencial en Salud Mental, SPAIN

## Abstract

**Background:**

This therapeutic drug monitoring (TDM) study aimed to determine the role of olanzapine (OLZ) and *N*-desmethyl-OLZ (DMO) levels in the therapeutic efficacy of OLZ in patients with schizophrenia.

**Method:**

Plasma concentrations of OLZ (C_OLZ_) and DMO (C_DMO_) in schizophrenic patients 12 hours post-dose were assessed. The correlations of C_OLZ_ and C_DMO_ with the various scores of the Positive and Negative Syndrome Scale (PANSS) were evaluated. A receiver operating characteristic curve (ROC) was utilized to identify the threshold C_OLZ_ and C_OLZ_/C_DMO_ ratio for maintenance of satisfactory efficacy.

**Results:**

A total of 151 samples from patients with schizophrenia were analyzed for individual C_OLZ_ and C_DMO_ levels. The mean C_OLZ_ and C_DMO_ levels were 37.0 ± 25.6 and 6.9 ± 4.7 ng/mL, respectively, and C_OLZ_ was ~50% higher in female or nonsmokers (p<0.01). In all patients, the daily dose of OLZ was positively correlated with C_OLZ_ and C_DMO_. Linear relationships between C_OLZ_ and OLZ dose were observed in both nonsmokers and smokers (r_s_ = 0.306, 0.426, p<0.01), although C_DMO_ was only correlated with OLZ dose in smokers (r_s_ = 0.485, p<0.01) and not nonsmokers. In all patients, C_OLZ_ was marginally negatively correlated with the total PANSS score. The total PANSS score was significantly negatively correlated with the C_OLZ_/C_DMO_ ratio (p<0.005), except in smokers. The ROC analysis identified a C_OLZ_/C_DMO_ ratio ≥2.99 or C_OLZ_ ≥22.77 ng/mL as a predictor of maintenance of an at least mildly ill status (PANSS score ≤58) of schizophrenia in all patients.

**Conclusions:**

A significantly negative correlation between the steady-state C_OLZ_/C_DMO_ ratio and total PANSS score was observed in Taiwanese schizophrenic patients. TDM of both OLZ and DMO levels could assist clinical practice when individualizing OLZ dosage adjustments for patients with schizophrenia.

## Introduction

Schizophrenia is a chronic and disabling mental disease [1]. Two generations of antipsychotic drugs are used for schizophrenia symptom management. The CATIE (Clinical Antipsychotic Trials for Intervention Effectiveness) Schizophrenia Trial examined fundamental issues about second-generation antipsychotic medications. Atypical antipsychotics, such as clozapine, olanzapine (OLZ), and quetiapine, have fewer extrapyramidal side effects but are associated with weight gain and metabolic problems [2]. The World Federation of Societies of Biological Psychiatry suggests OLZ, quetiapine, and risperidone as first-line medications of first-episode schizophrenia patients [3]. Moreover, Hatta reported that OLZ and risperidone are superior to quetiapine and aripiprazole for the acute treatment of psychosis in hospitalized patients experiencing their first episode [4]. A more recent study demonstrated that OLZ was more likely to be reserved for patients with more severe schizophrenia symptoms, whereas OLZ was less likely to be prescribed to patients with heavier body weight and those with a higher BMI [5]. However, the CATIE Schizophrenia Trial found that olanzapine is relatively the most effective antipsychotics, as measured by treatment discontinuation [2]. The olanzapine’s superiority in efficacy needs to be weighed against weight gain and metabolic abnormalities than other second generation antipsychotics [6].

OLZ is a thienobenzodiazepine (2-methyl-4-(4-methyl-1-piperazinyl) -10H-thieno[2,3-b][1,5]benzodiazepine) with high affinity for various receptors [7,8]. Indications for OLZ include schizophrenia, mania and maintenance of bipolar disorders [9]. The pharmacokinetic parameters of OLZ indicate that OLZ levels are linearly correlated with dose, with 60% bioavailability and ~93% protein binding in blood [10]. The main components present in blood are OLZ-10-N-glucuronide and 4’-N-desmethyl-olanzapine (DMO), which is formed by the metabolism of OLZ by cytochrome P450 (CYP) 1A2 [8,10,11]. Smoking status, sex, and race accounted for 26%, 12%, and 7% of the variability of olanzapine clearance, respectively [12]. The plasma concentration of OLZ (C_OLZ_) increases linearly with increasing daily oral doses and is correlated with improvements in the clinical symptoms of schizophrenia patients [13–15]. According to the guidelines for therapeutic drug monitoring (TDM) in psychiatry, C_OLZ_ is suggested to be within 20–80 ng/mL at 12 hours after dosing for patients with schizophrenia [16]. Perry et al. reported that patients with C_OLZ_ higher than 23.2 ng/mL at 12 hours after dosing exhibit clinical responses to OLZ therapy [14]. A minimum effective concentration of 9.3 ng/mL (24 hours post-dose) has also been reported [17]. The Maudsley prescribing guidelines [18] note that toxicity can be induced at C_OLZ_ higher than 100 ng/mL and that there is a risk of death when levels reach 160 ng/mL.

To ensure effectiveness and minimize the side effects of OLZ, the AGNP Consensus Guidelines suggest that patients receiving OLZ treatment may benefit from TDM [16] because C_OLZ_ exhibits inter-individual variations of up to 25-fold [19]. Several non-genetic factors, such as age, gender, smoking, co-medication or disease states, may influence OLZ levels [20]. Information about the role of DMO concentrations (C_DMO_) in the clinical efficacy of OLZ is scare.

Therefore, this study aimed to investigate the roles of C_OLZ_ and C_DMO_ in OLZ effectiveness.

## Materials and Methods

This study used modified high-performance liquid chromatography (HPLC) coupled with electrochemical detector as described in our previous study [21], except that 80 mM phosphate buffer (NaH_2_PO_4_) was used to enhance system stability and LC-MS-grade acetonitrile was used to minimize noise. Patients meeting the inclusion criteria (aged 18–60 years, stable OLZ dose for at least three months, and full capacity to consent) were recruited according to the methodology of Lu et al. [21]. This study, including its procedures, was approved by the institutional review board and the ethics committee of Taipei Medical University (Approval No. F950206), and all clinical investigations were conducted according to the principles expressed in the Declaration of Helsinki. The participants were included in the study only if they had full capacity by themselves to provide written consent to participate in the study. The understanding of the patients of all procedures and their capacity to provide consent was assessed by direct examination of the participants by a clinician experienced in the evaluation of mental illness. Patients with addictions and who were pregnant, lactating, or had disease conditions that might interfere with monitoring were excluded. Samples from patients prescribed medications with evidence for interactions with OLZ were excluded. Samples were drawn in the morning approximately 12 hours after the last dose of OLZ and analyzed to determine C_OLZ_ and C_DMO_.

During the study period, the symptom severity of the recruited patients was also routinely assessed by a psychiatric physician using the Positive and Negative Syndrome Scale (PANSS). PANSS is clinically used to assess schizophrenic symptoms, including positive, negative and general psychopathology scales [22]. The clinical efficacy of olanzapine was determined using the PANSS [23,24]. PANSS scores ≤58 were defined as mildly ill, as established by Leucht and coworkers[23].

Descriptive statistics are presented as the mean ± standard deviation (SD). The drug concentration normalized by the administered dose is expressed as the C/D ratio. Recruited patients were subgrouped by smoking status and sex, which are known to influence variations of OLZ levels. Intergroup comparisons were performed by Mann-Whitney U test. To quantify the ability of drug-level indicators (C_OLZ_, C_DMO_ or ratio of C_OLZ_/C_DMO_) to identify schizophrenic symptomatic status in terms of various PANSS scores, Spearman’s rank order correlation method (r_s_) analysis was conducted using SigmaPlot 12.0. The false discovery rate (FDR) was applied for multiple testing corrections [25]. To determine the cut-off values to indicate that the patients’ clinical symptoms were maintained at an at least mildly ill status (PANSS total score ≤58) [23], the ROC curve was plotted using SPSS 20, and the area under the curve (AUC) was used for accuracy comparison. The indicators with the highest sensitivity and specificity were considered the thresholds for each test. A p value <0.05 was considered significant.

## Results

### Demographic Characteristics and Subgroup Analysis of Drug Concentration Indicators

This study included a total of 151 samples from patients with schizophrenia. No patients had previously been administered clozapine, and therefore the use of clozapine as an internal standard did not interfere with the reliability of the bioanalysis. Demographic characteristics and steady-state drug level indicators are presented in Table 1. There were no significant gender differences in demographic characteristics except body weight. Because smoking status and gender can influence the variation in drug levels, patients were subgrouped for comparison. There were no differences in DMO levels or C/D ratio between males and females. Compared with male patients, females had higher OLZ levels and C/D ratios (p<0.001), whereas the mean administered doses were similar. OLZ levels, OLZ C/D ratios, and C_OLZ_/C_DMO_ ratios were lower in smokers than in nonsmokers (p<0.05), but OLZ doses were similar in the two groups. Smokers represented 46.48% of the male and 8.75% of the female patients.

**Table 1:** Demographic characteristics.

Parameters	All	Smoker	Non-smoker	*p* value *	Male	Female	*p* value $
	(n = 151)	(n = 40)	(n = 111$)		(n = 71)	(n = 80)	
**Age (y/o)**	41.3±12.1	40.8±12.6	41.5±12.0	0.914	40.1±11.9	42.4±12.1	0.350
**OLZ dose (mg)**	14.2±5.4	15.5±5.4	13.7±5.4	0.063	14.5±5.7	13.9±5.2	0.454
**Weight (kg)**	68.1±15.1	71.6±14.5	66.8±15.1	0.050	72.9±15.8	63.8±13.0	<0.001
**BMI (kg/m2)**	25.9±6.4	26.0±9.0	25.9±5.2	0.372	26.1±7.7	25.8±4.9	0.729
**PANSS, Total**	57.2±16.4	58.4±15.7	56.8±16.7	0.467	57.6±15.8	56.8±17.0	0.645
Positive	15.2±5.3	15.3±4.3	15.1±5.6	0.775	15±5.3	15.3±5.4	0.551
Negative	15.0±5.8	15.7±6.0	14.8±5.7	0.297	15.7±5.9	14.3±5.6	0.098
General	27.0±8.2	27.4±8.2	26.9±8.2	0.707	26.9±7.6	27.2±8.7	0.982
**DMO level (ng/mL)**	6.9±4.7	7.6±6.3	6.6±4.0	0.763	7.4±5.7	6.4±3.7	0.505
**OLZ level (ng/mL)**	37.0±25.6	27.0±23.1	40.5±25.6	0.004	29.3±24.0	43.8±25.1	<0.001
**DMO C/D (ng/mL/mg)**	0.6±0.4	0.5±0.4	0.6±0.4	0.559	0.6±0.4	0.5±0.4	0.376
**OLZ C/D (ng/mL/mg)**	2.9±2.3	1.7±1.2	3.3±2.5	<0.001	2.1±1.7	3.5±2.6	<0.001
**Ratio of OLZ/DMO**	7.0±6.16	4.8±4.1	7.85±6.4	0.005	5.4±4.9	8.6±6.6	<0.001

Abbreviations: OLZ, olanzapine; DMO, N-desmethyl-olanzapine; BMI, body mass index

*smoker vs. nonsmoker;

^**$**^female vs. male;

*p* value was set at 0.05; an independent t-test was used for age, and a Mann-Whitney U-test was used for all other comparisons.

### Correlation Analysis of Drug-Level Indicators and OLZ Dose

**Fig 1:**
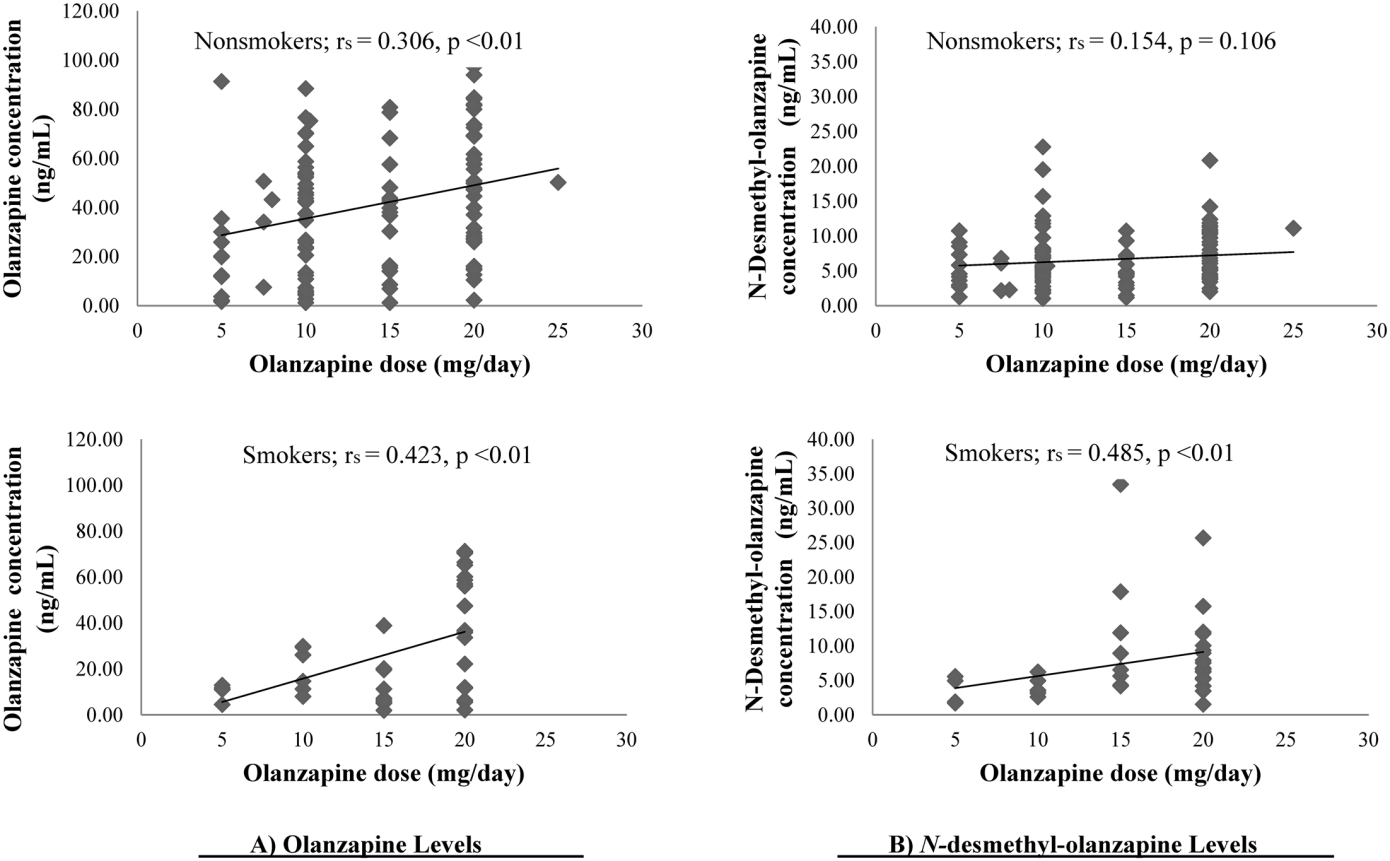
Relationships between drug levels and olanzapine dose among nonsmokers and smokers. A) olanzapine; B) N-desmethyl-olanzapine.

Daily doses of OLZ in all patients were positively correlated with C_OLZ_ (r_s_ = +0.300, p<0.001) and C_DMO_ (r_s_ = +0.254, p<0.001). Significant dose-concentration correlations for OLZ were observed in nonsmokers (r_s_ = +0.306, p<0.01) and smokers (r_s_ = +0.423, p<0.01) (Fig 1A). No correlation was observed between OLZ dose and DMO levels in nonsmokers, but OLZ dose and C_DMO_ were significantly correlated in smokers (r_s_ = +0.485, p<0.01) (Fig 1B). The C_OLZ_/C_DMO_ ratio was not correlated with OLZ dose.

### Correlation Analysis of Drug Level Indicators and PANSS Scores

**Fig 2:**
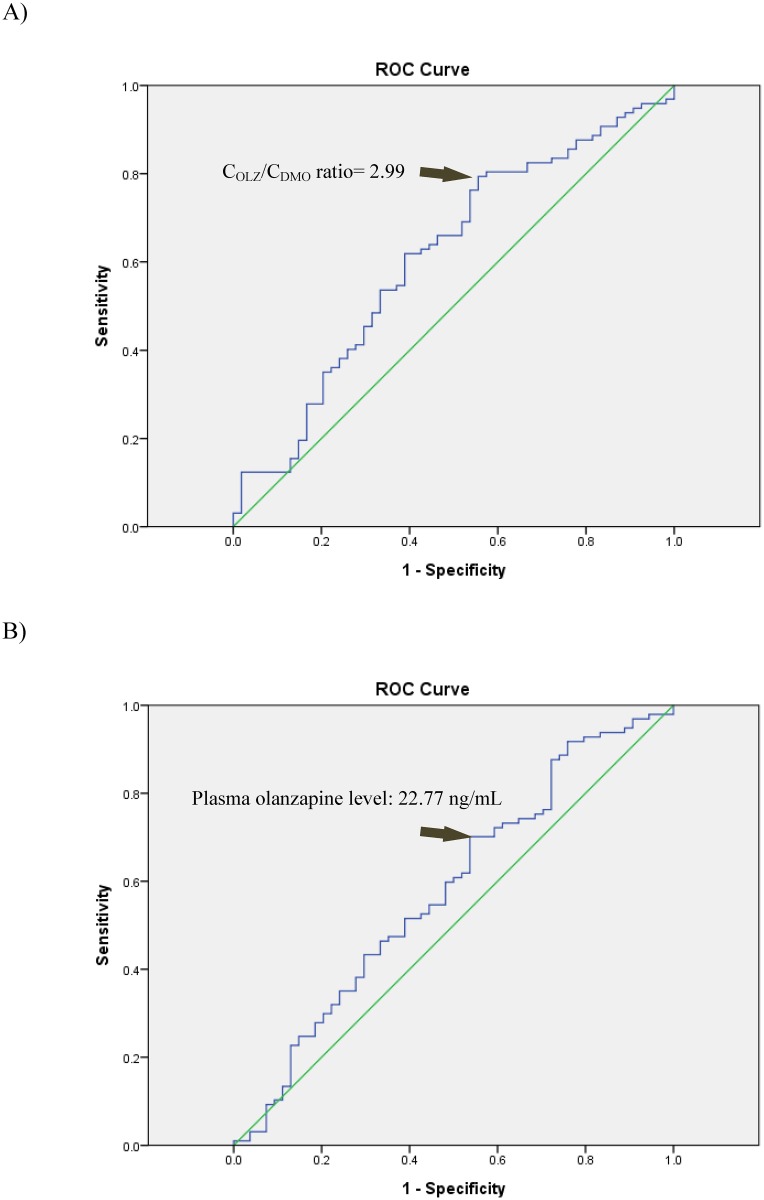
Receiver operating characteristic curves for A) the C_OLZ_/C_DMO_ ratio and B) plasma olanzapine levels as predictors of clinical symptoms maintained at an at least mildly ill status (PANSS score ≤58) among the recruited patients (n = 151).

**Fig 3:**
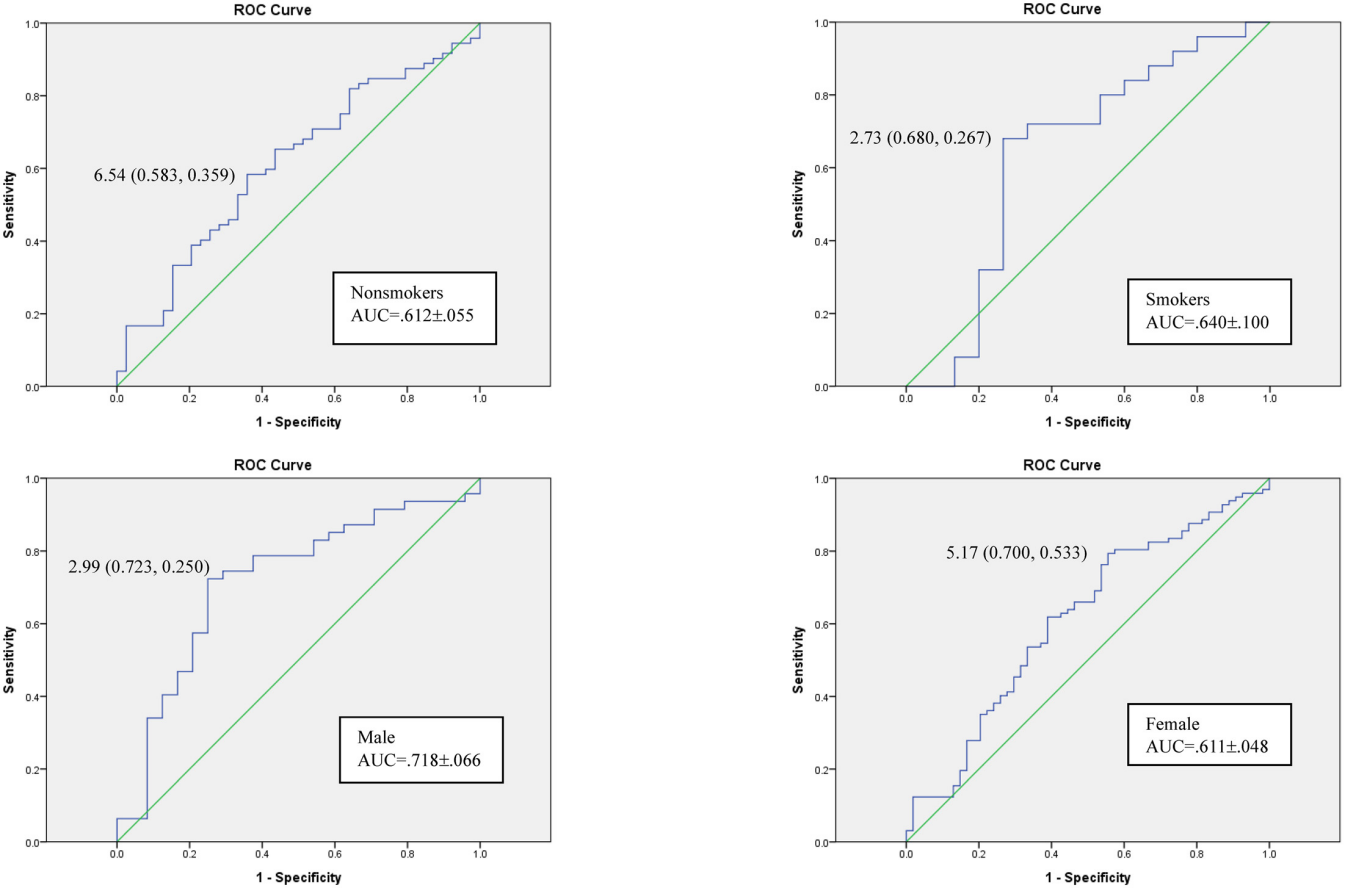
Receiver operating characteristic curves for the C_OLZ_/C_DMO_ ratio cut-off values as predictors of maintaining an at least mildly ill status (PANSS score ≤58) for the various subgroups of patients.

Correlation analysis was performed to determine if C_OLZ_, C_DMO_, or the C_OLZ_/C_DMO_ ratio was more reliable as a predictor of the PANSS score (Table 2). The total PANSS score was significantly negatively correlated with the C_OLZ_/C_DMO_ ratio (p<0.01) in the whole sample and in non-smokers but not in smokers. To determine the cut-off values of drug levels to indicate that the patients’ symptoms were maintained at an at least mildly ill status (PANSS score ≤58) and to determine if the C_OLZ_/C_DMO_ ratio can be used to predict symptomatic status beyond correlational analysis, ROC analysis was performed. A C_OLZ_/C_DMO_ ratio greater than 2.99 (AUC = 0.611±0.048; sensitivity: 0.794; specificity: 0.444; accuracy: 66.9%) and C_OLZ_ greater than 22.77 ng/mL (AUC = 0.579±0.049; sensitivity: 0.701; specificity: 0.463; accuracy: 61.6%) were identified as predictors of symptomatic status in all patients (Fig 2). The ROC subgroup analyses for nonsmokers, smokers, and male or female patients are presented in Fig 3.

**Table 2:** Correlation tests for PANSS scores and levels of olanzapine and its metabolite DMO.

All (n = 151)	DMO	OLZ	OLZ/DMO
**PANSS, total**			
***rs* =**	0.125	-0.174*	-0.250*¶
***p* =**	0.127	0.0328	0.00201
**PANSS, positive**			
***rs* =**	0.0369	-0.152	-0.188*
***p* =**	0.652	0.0623	0.0211
**PANSS, negative**			
***rs* =**	0.178*	-0.0206	-0.148
***p* =**	0.0285	0.802	0.0696
**PANSS, general**			
***rs* =**	0.0651	-0.240*¶	-0.244*¶
***p* =**	0.427	0.00301	0.00256
**Nonsmokers (n = 111)**			
**PANSS, total**			
***rs* =**	0.175	-0.143	-0.289*¶
***p* =**	0.0658	0.135	0.00219
**PANSS, positive**			
***rs* =**	0.113	-0.0861	-0.184
***p* =**	0.236	0.368	0.0527
**PANSS, negative**			
***rs* =**	0.195*	0.012	-0.190*
***p* =**	0.04	0.901	0.0456
**PANSS, general**			
***rs* =**	0.116	-0.197*	-0.254*
***p* =**	0.227	0.038	0.00737
**Smokers (n = 40)**			
**PANSS, total**			
***rs* =**	-0.0434	-0.286	-0.162
***p* =**	0.789	0.0732	0.315
**PANSS, positive**			
***rs* =**	-0.251	-0.407*	-0.202
***p* =**	0.117	0.00945	0.209
**PANSS, negative**			
***rs* =**	0.127	-0.0924	-0.0689
***p* =**	0.433	0.568	0.671
**PANSS, general**			
***rs* =**	-0.103	-0.399*	-0.239
***p* =**	0.523	0.011	0.137

*p value <0.05; Spearman’s rank order correlation method.

^**¶**^*r*_*s*_ remained significant after FDR correction.

Abbreviations: OLZ, olanzapine; DMO, *N*-desmethyl-olanzapine; C/D ratio, concentration-dose ratio; PANSS, Positive and Negative Syndrome Scale

## Discussion

Among all samples, the OLZ dose was linearly correlated with C_OLZ_ and C_DMO_, similar to Skogh’s results [19]. The present study primarily explored the application of the C_OLZ_/C_DMO_ ratio in monitoring clinical symptomatic status as indicated by PANSS scores in patients with schizophrenia. The C_OLZ_/C_DMO_ ratio was significantly negatively correlated with PANSS scores (total and general psychopathology) after FDR correction. The role of the C_OLZ_/C_DMO_ ratio in predicting clinical symptomatic status was then assessed using ROC analysis. The C_OLZ_/C_DMO_ ratio threshold was 2.99 and yielded superior diagnostic accuracy compared to C_OLZ_.

According to the AGNP-TDM guidelines [16], C_OLZ_ is suggested to be within the therapeutic range (20~80 ng/mL) for symptom improvement because intra-individual differences can reach 29.7-fold, and levels vary by gender and smoking behavior [26]. The mean C_OLZ_ values of the current samples or subgroups fall within the suggested therapeutic ranges. As shown in Fig 2B, overall, 70% (68/97) of samples with OLZ levels higher than 22.77 ng/mL met the criteria for mildly ill status following OLZ therapy. However, C_OLZ_ failed to predict the clinical symptomatic status of female patients (AUC of the ROC analysis was less than 0.5).

The cut-off level of OLZ (22.77 ng/mL) identified in this study is similar to previously reported response levels (23.2 ng/mL) for acutely ill patients or stably treated patients [14,27]. However, an improved total Brief Psychiatric Rating Scale (BPRS) score is not correlated with the plasma OLZ concentration, although individual BPRS scores related to improvement of suspiciousness, hallucinations, and blunted affect are significantly correlated with plasma OLZ concentration [15]. In a trial in a Western population, changes in PANSS scores during periods of OLZ therapy were not correlated with C_OLZ_ [28]. Similarly, in this study in a Taiwanese population, OLZ levels were not correlated with PANSS scores (except the general psychopathology score) in all samples or subgroup analyses of nonsmokers/smokers after FDR correction. By contrast, the C_OLZ_/C_DMO_ ratio was correlated with total and general PANSS scores for all patients, and the C_OLZ_/C_DMO_ ratio in nonsmokers was also correlated with the total PANSS score (p<0.005).

C_OLZ_ or its C/D ratio was higher in females, consistent with the previous literature [19,20,28]. These gender differences may be related to higher CYP1A2 activity in males [19]. Moreover, lean body mass also causes gender differences because females commonly have more fatty tissue in which OLZ may accumulate, resulting in increased drug levels [29]. No differences in C_DMO_ or C_DMO_/D ratios were observed between genders, consistent with Skogh’s results [19]. However, female nonsmokers had significantly higher C_DMO_/D ratios than female smokers (0.56 vs. 0.29). The absence of differences in the various drug level indicators between male smokers and nonsmokers may have occurred because the male smokers were light cigarette smokers who smoked fewer than 4 cigarettes per day [30].

OLZ levels may have been higher in nonsmokers (Table 1) due to gender effects because 65.8% of the samples from nonsmokers were obtained from females. OLZ levels were nearly 1.42 times higher in female nonsmokers than in male nonsmokers, whereas DMO levels were similar. Accordingly, the threshold ratio for satisfactory efficacy was nearly two times higher for nonsmokers or females (8.8% smokers) than for smokers or males (82.5% smokers). Females may have lower metabolic enzyme activity [15], and smoking cigarettes results in greater induction of CYP1A2 activity, thus resulting in larger differences between female nonsmoker vs. female smoker in the C_OLZ_/D ratio (3.66 vs. 1.9). Similar observations are also reported by a previous study [31] in children and adolescents which reported that male patients displayed a lower C_OLZ_/C_DMO_ ratio than females.

The drug levels in this study were measured at the steady-state 12-hr post-dose. The DMO and OLZ levels can both be considered averages of the steady-state plasma concentrations as described in a pharmacokinetic study [32]. In the subgroup analysis (Fig 3), the cut-off values of the C_OLZ_/C_DMO_ ratio for the subgroups of males or smokers had higher sensitivity and specificity than the values for the other subgroups and thus may be more reliable. However, these results were simply resolved from cross-sectional clinical scores; future studies should utilize changes in symptomatic scores from baseline in a larger population to provide a good guide for clinical response.

In conclusion, a significantly negative correlation between the steady-state C_OLZ_/C_DMO_ ratio and total PANSS score was observed in Taiwanese schizophrenic patients. The C_OLZ_/C_DMO_ ratio can assist in determining individual metabolic differences due to smoking status and gender. Measuring 12-hr post-dose levels of DMO and OLZ in clinical practice may be employed to optimize treatment outcomes that are less than satisfactory. Our findings suggest that TDM of both OLZ and DMO is useful to assess efficacy in patients treated with OLZ.
